# Proteinuria can predict prognosis after liver transplantation

**DOI:** 10.1186/s12893-016-0176-8

**Published:** 2016-09-15

**Authors:** Heng-Chih Pan, Ying-Jen Chen, Jhe-Ping Lin, Ming-Jung Tsai, Chang-Chyi Jenq, Wei-Chen Lee, Ming-Hung Tsai, Pei-Chun Fan, Chih-Hsiang Chang, Ming-Yang Chang, Ya-Chung Tian, Cheng-Chieh Hung, Ji-Tseng Fang, Chih-Wei Yang, Yung-Chang Chen

**Affiliations:** 1 Department of Nephrology, Chang Gung Memorial Hospital, Keelung, Taiwan; 2 Division of General Internal Medicine and Geriatrics Medicine, Chang Gung Memorial Hospital, Taipei, Taiwan; 3 Chang Gung University College of Medicine, Taoyuan, Taiwan; 4 Kidney Research Center, Department of Nephrology, Chang Gung Memorial Hospital, Taipei, Taiwan; 5 Laboratory of Immunology, Department of General Surgery, Chang Gung Memorial Hospital, Taipei, Taiwan; 6 Division of Gastroenterology, Chang Gung Memorial Hospital, Taipei, Taiwan

**Keywords:** Liver transplantation, Proteinuria, SOFA, Prognosis, Mortality

## Abstract

**Background:**

Proteinuria is a manifestation of renal dysfunction and it has been demonstrated to be a significant prognostic factor in various clinical situations. The study was designed to analyze prognosis of patients receiving liver transplantation as well as to determine predictive performance of perioperative proteinuria.

**Methods:**

We retrospectively reviewed data of patients who had received a liver transplant in a medical center between 2002 and 2010. Demographic information and clinical characteristic parameters were recorded on the day of intensive care unit admission before operation and on postoperative days 1, 7, and 14.

**Results:**

Among a total of 323 patients, in-hospital mortality and 90-day mortality rates were 13.0 % (42/323) and 14.2 % (46/323), respectively. Patients with proteinuria on admission had higher rates of acute kidney injury (26.8 % vs. 8.8 %, *p* < 0.001), severe infection episodes (48.8 % vs. 30.7 %, *p* = 0.023), hospital death (31.1 % vs. 10.1 %, *p* < 0.001), and 90-day mortality (37.7 % vs. 10.9 %, *p* < 0.001). Multivariate analysis showed that proteinuria on admission and Sequential Organ Failure Assessment (SOFA) score were independent predictors of in-hospital mortality. The discriminatory ability of proteinuria plus SOFA was even better than that of SOFA alone, especially on postoperative day 1.

**Conclusions:**

The presence of proteinuria before liver transplantation is supposed to be recognized as a negative predictor for in-hospital survival. Moreover, the presence of proteinuria after liver transplantation can assist in the early prediction of poor short-term prognosis for patients receiving liver transplantation.

## Background

Proteinuria has been suggested to be a predictive factor and an important tool for differentiating the etiology of renal dysfunction in various clinical scenarios [1, 2]. The good predictive performance of preoperative proteinuria utilized for the development of renal failure after operation has been reported [3]. Lin et al. also demonstrated that the presence of proteinuria in patients with end-stage liver disease is associated with increased risk of intensive care unit (ICU) mortality and poor short-term outcome [4].

In the literature, prognostic significance of several scoring systems for end-stage liver disease has been validated [5–7]. Wong and colleagues further compared the predictive accuracy of the commonly used scores in 149 end-stage liver disease patients undergone liver transplantation. The Sequential Organ Failure Assessment (SOFA) system was found to be superior to Child-Pugh points (CP points) and Model for End-Stage Liver Disease (MELD) score, and postoperative day 7 SOFA had the best discriminative power for predicting 3-month and 1-year mortality after liver transplantation [8].

Renal dysfunction is one of the most significant adverse events in patients awaiting or undergoing a liver transplant, and its occurrence generally indicates a high rate of poor prognosis [9, 10]. Prediction of acute kidney injury (AKI) is important for clinical decision making. Numerous researches have demonstrated that the presence of proteinuria provides a clue to the structural impairment of the kidney and reflects increased risk of developing AKI in general population [11, 12]. In spite of the fact that proteinuria has been increasingly considered as a significant manifestation of acute or chronic renal disease [13], no study clarify the association between presence of proteinuria and prognosis of patients undergoing liver transplant. This study aims to assess proteinuria as an early marker of renal dysfunction for liver transplant as well as to compare the outcome prediction efficacy of proteinuria with that of the main scores in the setting of liver transplant.

## Methods

### Patient information and data collection

This research was performed from October 2002 to December 2010 in a 2000-bed medical center in Taiwan. A total of 323 patients with end-stage liver disease and acute liver failure received liver transplant were included. Patients less than 18 years of age, patients who had previously received liver transplant, and patients with end-stage renal disease were excluded.

We retrospectively reviewed following data: demographic information, aetiologies of primary liver disease, clinical parameters, donor type, anesthesia time, operation time, duration of hospitalization and ICU stay, and outcome. Illness severity was evaluated on the day of ICU admission before operation and on postoperative day 1, 7, and 14. The primary outcome of this research was in-hospital mortality rate. Follow-up at 90-day after transplantation was performed by chart records review or telephone interview [8].

### Definitions

Urine dipstick analysis was used to detect proteinuria. The results were graded as negative (less than 15 mg/dL), trace (15 to 30 mg/dL), 1+ (30 to 100 mg/dL), 2+ (100 to 300 mg/dL), 3+ (300 to 1000 mg/dL) or 4+ (more than 1000 mg/dL). In this study, proteinuria was defined as presence (urine dipstick reading trace, or ≥ 1+) or absence (negative urine dipstick reading). The urinary analysis was performed on ICU admission and postoperative days 1, 7, and 14 [4].

Severity of liver disease was assessed by CP points and MELD score [14, 15]. Severity of illness was also graded by SOFA score according to the six organ systems. AKI was diagnosed according to definition of the RIFLE criteria (Table 1). The RIFLE classification was also used to classify patients into risk (RIFLE-R), injury (RIFLE-I), and failure (RIFLE-F) groups [16]. No patient met the criteria for classification of loss of kidney function (RIFLE-L) or end-stage renal disease (RIFLE-E). The following simple model for mortality was constructed: non–acute kidney injury (0 points), RIFLE-R (1 point), RIFLE-I (2 points), and RIFLE-F (3 points) [15]. The worst values measured on the day of ICU admission and postoperative days 1, 7, and 14 were recorded.

**Table 1:** The criteria of SOFA score and RIFLE classification

SOFA Score	0	1	2	3	4
Respiration					
PaO2/FiO2	>400	>300–≤ 400	>200–≤ 300	>100–≤ 200 with ventilator	≤100 with ventilator
Coagulation					
Platelets, ×10^3^/mm^3^	>150	>100–≤ 150	>50–≤ 100	>20–≤ 50	≤20
Liver					
Bilirubin, mg/dL (μmol/L)	<1.2 (<20)	≥1.2–< 2.0 (20–32)	≥2.0–< 6.0 (33–101)	≥6.0–< 12.0 (102–204)	≥12.0 (>204)
Cardiovascular					
Hypotension	MAP ≥ 70 mm Hg	MAP <70 mm Hg	Dopamine ≤5 or dobutamine (any dose)^a^	Dopamine >5 or epi ≤0.1 or norepi ≤0.1^a^	Dopamine >15 or epi >0.1 or norepi >0.1^a^
CNS					
Glasgow Coma Score	15	13–14	10–12	6–9	<6
Renal					
Creatinine, mg/dL (μmol/L) or urine output	<1.2 (<110)	≥1.2–< 2.0 (110–170)	≥2.0–< 3.5 (171–299)	≥3.5–< 5.0 (300–440) or <500 mL/day	≥5.0 (>440) or <200 mL/day
RIFLE Classification	SCr criteria				UO Criteria
Definition	SCr changes over 1–7 days, sustained for more than 24 h				UO < 0.5 ml/kg/h × 6 h
Risk	Increase in SCr ≥ 1.5 × baseline or decrease in GFR ≥ 25 %				UO < 0.5 ml/kg/h × 6 h
Injury	Increase in SCr ≥ 2.0 × baseline or decrease in GFR ≥ 50 %				UO < 0.5 ml/kg/h × 12 h
Failure	Increase in SCr ≥ 3.0 × baseline or an absolute serum creatinine ≥ 4.0 mg/dl with an acute rise of at least 0.5 mg/dl or decrease in GFR ≥ 75 %				UO < 0.5 ml/kg/h × 24 h or anuria × 12 h
Loss	Complete loss of kidney function > 4 weeks				
ESRD	End-stage renal disease (>3 months)				

^a^Abbreviations: *SOFA* the sequential organ failure assessment, *RIFLE* risk of renal failure, injury to kidney, failure of kidney function, loss of kidney function, and end-stage renal failure, *SCr* serum creatinine, *UO* urine output, *hrs* hours, *ESRD* end-stage renal disease, *RRT* renal replacement therapy

### Statistical analysis

Data analyses were performed using the statistical package SPSS 19.0 (SPSS, Inc., Chicago, IL, USA). All statistical tests are 2-tailed. A *p-* value of <0.05 is considered to represent statistical significance. Continuous variables were presented as means and standard derivations, and categorical data were summarized as frequency and percentage unless otherwise stated. Hospital survivors were compared with nonsurvivors in the primary analysis. Kolmogorov–Smirnov test was employed for testing normal distribution. Normally distributed continuous variables were compared by Student’s *t*-test and non-normally distributed ones were compared by Mann–Whitney *U* test. Categorical data were tested by the chi-square test. The risk factors for in-hospital mortality were assessed by univariate analysis, and statistically significant variables were included in the multivariate analysis. For analyzing these variables, backward multiple logistic regression model was employed.

Hosmer–Lemeshow goodness-of-fit test was used to examined calibration and compare the number of predicted and observed mortality. Discrimination in predicting 90-day mortality was assessed by area under the receiver operating characteristic (AUROC) curve. Nonparametric approach was used to compare the AUROC values. Analyses of the ROC curves were applied for calculating sensitivity, specificity, and overall correctness. The cutoff value was decided according to the ability to offer the highest Youden index [17]. Cumulative survival curves were plotted using the Kaplan-Meier method and compared by the log rank test. Correlation of proteinuria and serum creatinine (SCr) measured on ICU admission was measured by Pearson analysis and linear regression. The prevalence of proteinuria before operation, and on postoperative day 1, 7, and 14 were compared between 90-day survivors and nonsurvivors by repeated-measures analysis of variance using the general linear model procedure.

## Results

### Patient characteristics

Three hundred and twenty-three patients who received liver transplant from October 2002 to December 2010 were enrolled. Overall in-hospital mortality rate was 13.0 % (42/323). Table 2 listed patient data and clinical characteristics of both survivors and non-survivors. Mean patient age was 51 years; 231 were male (71 %) and 92 were female (29 %). Ninety-one patients (28.2 %) received deceased-donor grafts. Mean length of ICU stay was 21 days. There was no significant difference in age or gender between survivors and non-survivors. Table 3 listed primary liver diseases and presumptive causes of AKI on the first day after transplantation. In this research, hepatitis B virus infection (34 %) was the leading cause of liver diseases, followed by hepatitis B virus infection with hepatoma (15 %). Patient who developed AKI tended to attribute to multiple reasons (23 %), followed by infection (13 %).

**Table 2:** Patient demographic data and clinical characteristics according to in-hospital mortality

	All patients (*n* = 323)	Survivors (*n* = 281)	Non-survivors (*n* = 42)	*P*-value
Age (years)	50.8 ± 10.4	50.9 ± 9.8	50.3 ± 13.8	NS (0.753)
Gender (M/F)	231/92	199/82	32/10	NS (0.583)
BMI on admission (kg/m^2^)	24.3 ± 4.0	24.7 ± 4.0	21.1 ± 2.4	**<0.001**
History of diabetes mellitus (yes/no)	55/268	46/235	9/33	NS (0.387)
History of chronic kidney disease (yes/no)	31/292	22/259	9/33	**0.005**
Proteinuria on admission (yes/no)	45/278	31/250	14/28	**<0.001**
Hemoglobin on admission (g/dL)	10.6 ± 2.2	10.7 ± 2.2	9.8 ± 2.1	**0.008**
Leukocytes on admission (× 10^9^/L)	2.9 ± 3.7	2.8 ± 3.5	3.3 ± 4.9	NS (0.569)
Platelets on admission (× 10^9^/L) [median]	72.7 [60]	72.9 ± [60]	71.1 [59]	NS (0.809)
Prothrombin time INR on admission	1.8 ± 0.7	1.8 ± 0.7	1.9 ± 0.7	NS (0.050)
Serum sodium on admission (mmol/L)	137.8 ± 5.7	137.9 ± 5.4	137.0 ± 8.0	NS (0.471)
AST on admission (U/L) [median]	88.8 [62]	87.5 [64]	98.3 [51]	NS (0.498)
ALT on admission (U/L) [median]	67.2 [39]	67.4 [40]	65.8 [34]	NS (0.938)
Total bilirubin on admission (μmol/L) [median]	145.4 [51]	130.0 [50]	244.5 [96]	**0.003**
A-a gradient on admission (mmHg) [median]	25.1 [17]	22.8 [17]	43.3 [18]	**0.039**
Serum creatinine on admission (μmol/L) [median]	97.2 [77]	92.2 [75]	114.9 [84]	NS (0.064)
Hepatorenal syndrome, n (%)	29 (9)	22 (8)	7 (17)	NS (0.079)
MAP on admission (mmHg)	86.1 ± 12.4	86.3 ± 12.7	84.7 ± 10.3	NS (0.427)
Child-Pugh points on admission	10.1 ± 2.6	9.9 ± 2.7	11.1 ± 2.0	**0.010**
MELD score on admission	17.6 ± 9.0	17.1 ± 8.9	21.4 ± 9.7	**0.025**
RIFLE on admission (No AKI/Risk/Injury/Failure)	286/16/9/12	250/13/9/9	36/3/0/3	NS (0.449)
SOFA score on admission	5.1 ± 2.7	4.8 ± 2.5	6.7 ± 3.3	**0.001**
Anesthesia time during operation (hours)	12.1 ± 1.8	12.1 ± 1.9	12.4 ± 1.5	NS (0.362)
Donor type (DDLT /LDLT)	91/232	74/207	17/25	**0.018**
Total operative time (mins) [median]	687.1 [683]	685.6 [683]	697.2 [682]	NS (0.589)
Cold ischemia time (mins) [median]	85.0 [15]	74.8 [15]	166.4 [17]	NS (0.125)
Warm ischemia time (mins) [median]	128.2 [122]	126.3 [121]	141.1 [129]	NS (0.115)
Graft-to-recipient weight ratio (%)	1.0 ± 0.3	1.0 ± 0.3	1.1 ± 0.5	NS (0.125)
Blood loss volume during operation (ml) [median]	3034 [2000]	2672 [1840]	4430 [2000]	**0.014**
Length of ICU stay (days) [median]	21.0 [14]	19.2 [14]	33.6 [24]	**0.002**
Length of hospital stay (days) [median]	47.8 [38]	46.7 [38]	54.7 [44]	NS (0.215)

Values in bold are statistically significant (*P*-value < 0.05)

There were significant differences in BMI on admission, history of chronic kidney disease, presence of proteinuria on admission, hemoglobin on admission, total bilirubin on admission, A-a gradient on admission, Child-Pugh points on admission, MELD score on admission, SOFA score on admission, blood loss volume during operation, and length of ICU stay

Abbreviation: *M* male, *F* female, *ICU* intensive care unit, *MAP* mean arterial pressure, *INR* international normalized ratio, *AST* aspartate aminotransferase, *ALT* alanine aminotransferase, *DM* diabetes mellitus, *MELD* model for end-stage liver disease, *SOFA* sequential organ failure assessment, *AKI* acute kidney injury, *DDLT* deceased donor liver transplantation, *LDLT* living donor liver transplantation

**Table 3:** Primary liver diseases and presumptive causes of AKI after operation according to in-hospital mortality

	All patients	Survivors	Non-survivors	*P*-value
	*n* = 323	*n* = 281	*n* = 42	
Primary liver disease	323 (100)	281 (100)	42 (100)	
Alcoholic, n (%)	16 (5)	12 (4)	4 (10)	NS (0.254)
Hepatitis B, n (%)	111 (34)	100 (36)	11 (26)	NS (0.848)
Hepatitis C, n (%)	31 (10)	23 (8)	8 (19)	**0.003**
Hepatoma, n (%)	3 (1)	3 (1)	0 (0)	NS (1.000)
Alcoholic + hepatitis B, n (%)	21 (6)	15 (7)	2 (5)	NS (1.000)
Alcoholic + hepatitis C, n (%)	5 (2)	5 (2)	0 (0)	NS (1.000)
Alcoholic + hepatoma, n (%)	3 (1)	3 (1)	0 (0)	NS (1.000)
Hepatitis B + hepatitis C, n (%)	17 (5)	14 (5)	3 (7)	NS (0.723)
Hepatitis B + hepatoma, n (%)	49 (15)	43 (15)	6 (14)	NS (0.172)
Hepatitis C + hepatoma, n (%)	31 (10)	29 (10)	2 (5)	NS (0.134)
Alcoholic + hepatitis B + hepatoma, n (%)	2 (1)	1 (1)	1 (2)	NS (0.429)
Other causes, n (%)^a^	34 (10)	29 (10)	5 (12)	NS (0.787)
Presence of AKI after transplantation (Post-OP day1)	125 (39)	101 (36)	24 (57)	**0.011**
Prerenal type of AKI, n (%)	2 (1)	2 (1)	0 (0)	NS (1.000)
Infection related AKI, n (%)	42 (13)	31 (11)	11 (26)	**0.006**
Nephrotoxic agent exposure related AKI, n (%)	6 (2)	6 (2)	0 (0)	NS (0.601)
Mixed type and other causes of AKI, n (%)^b^	75 (23)	62 (22)	13 (31)	NS (0.236)

Values in bold are statistically significant (*P*-value < 0.05)

Hepatitis C virus infection was independently associated with in-hospital mortality

Presence of infection related AKI on the first day after transplantation was independently associated with in-hospital mortality

^a^Biliary cirrhosis, biliary sclerosis, autoimmune hepatitis, Wilson’s disease, polycystic liver disease, drugs, and unknown causes

^b^Multifactor related, ischemia/reperfusion injury, or unknown cause

### Risk factors for adverse outcomes

Table 4 listed the correlation of operation time and newly onset proteinuria after transplantation. Among patients who received deceased-donor grafts, those with newly onset proteinuria tended to have longer cold ischemia time. While in patients who received living-donor grafts, those with newly onset proteinuria tended to have longer warm ischemia time.

**Table 4:** Operation time according to newly onset proteinuria after transplantation

	Patients with no proteinuria on admission	Patients with newly onset proteinuria on post-OP day 1	Patients with no newly onset proteinuria on post-OP day 1	*P*-value
LDLT	*n* = 208	*n* = 65 (31.2 %)	*n* = 143 (68.8 %)	
Total operative time (mins) [median]	703.9 [695]	789.3 [811]	707.6 [691]	NS (0.087)
Cold ischemia time (mins) [median]	85.4 [14]	27.9 [15]	21.9 [13]	NS (0.659)
Warm ischemia time (mins) [median]	128.2 [130]	194.0 [182]	127.6 [133]	**0.004**
DDLT	*n* = 70	*n* = 47 (67.1 %)	*n* = 23 (32.9 %)	
Total operative time (mins) [median]	640.0 [633]	646.2 [653]	625.8 [632]	NS (0.876)
Cold ischemia time (mins) [median]	651.3 [583]	756.5 [634]	552.0 [541]	**0.039**
Warm ischemia time (mins) [median]	113.5 [91]	114.0 [90]	101.4 [90]	NS (0.737)

Values in bold are statistically significant (*P*-value < 0.05)

In the LDLT group, patients with newly onset proteinuria had significantly longer warm ischemia time

In the DDLT group, patients with newly onset proteinuria had significantly longer cold ischemia time

Abbreviation: *DDLT* deceased donor liver transplantation, *LDLT* living donor liver transplantation

The univariate analysis showed that 9 (Table 5) out of the 31 variables (Table 2) were good prognostic indicators for in-hospital mortality. On performing the multivariate analysis, we recognized presence of proteinuria and SOFA determined on the first day of ICU admission as having independent prognostic significance (Table 5). Regression coefficients of these variables were used to calculate the odds of death in each patient as follows:

**Table 5:** Variables showing prognostic significance for in-hospital mortality

Parameters	Beta Coefficient	Standard error	Odds ratios (95 % CI)	*P*-value
Univariate logistic regression				
BMI on admission (kg/m^2^)	−0.247	0.050	0.781 (0.708–0.861)	**<0.001**
History of chronic kidney disease	1.167	0.437	3.211 (1.364–7.557)	**0.008**
Proteinuria on admission	1.480	0.398	4.391 (2.011–9.587)	**<0.001**
Hemoglobin on admission (g/dL)	−0.218	0.083	0.804 (0.683–0.947)	**0.009**
Total bilirubin on admission (mg/dL)	0.037	0.011	1.038 (1.015–1.061)	**0.001**
Donor type (DDLT /LDLT)	−0.41	0.169	0.664 (0.477–0.925)	**0.015**
A-a gradient on admission	0.009	0.003	1.009 (1.002–1.016)	**0.007**
Child-Pugh points on admission	0.176	0.070	1.193 (1.040–1.368)	**0.012**
MELD score on admission	0.046	0.056	1.047 (0.999–1.098)	NS (0.056)
SOFA score on admission	0.219	0.057	1.245 (1.114–1.391)	**<0.001**
Blood loss volume during operation (ml)	<0.001	<0.001	1.000 (1.000–1.000)	**0.003**
Length of ICU stay (days)	0.018	0.006	1.018 (1.007–1.029)	**0.002**
Multivariate logistic regression				
Proteinuria on admission	1.320	0.478	3.745 (1.468–9.554)	**0.006**
SOFA on admission	0.157	0.067	1.170 (1.027–1.333)	**0.019**
Constant	−2.471	0.245	0.085	**<0.001**

On performing multivariate logistic regression, the presence of proteinuria on admission and SOFA score on admission had independent prognostic significance for assessing in-hospital mortality

Abbreviation: *MELD* model for end-stage liver disease, *SOFA* sequential organ failure assessment

Logarithm of odds of death = −2.471 + 1.320 × Proteinuria + 0.157 × SOFA score.

### Calibration and discrimination of the scoring systems

Table 6 showed values of calibration and discrimination of proteinuria, CP points, MELD, RIFLE, and SOFA in predicting 90-day mortality. For assessing calibration, the number of observed and predicted mortality was compared by Hosmer-Lemeshow goodness-of-fit. Discriminatory power was assessed by AUROC. On basis of the ROC analysis, discriminatory ability of SOFA and MELD determined on preoperative, postoperative days 1, 7, and 14 were better than that of CP points and proteinuria.

**Table 6:** Calibration and discrimination for the scoring methods used in predicting 90-day mortality

	Calibration			Discrimination		
	Goodness-of-fit (x^2^)	df	*p*	AUROC ± SE	95 % CI	*P*
On admission						
Proteinuria	-	-	-	0.582 ± 0.053	0.479–0.685	NS (0.100)
Child-Pugh points	10.157	7	0.180	0.580 ± 0.041	0.499–0.662	NS (0.087)
MELD score	5.845	8	0.665	0.609 ± 0.067	0.418–0.680	NS (0.067)
RIFLE	─	─	─	0.577 ± 0.067	0.355–0.618	NS (0.845)
SOFA	1.618	5	0.899	0.648 ± 0.049	0.552–0.745	**0.002**
Proteinuria plus SOFA	1.047	5	0.959	0.659 ± 0.052	0.557–0.761	**0.002**
Proteinuria plus nonrenal SOFA	6.347	6	0.385	0.658 ± 0.055	0.550–0.765	**0.002**
Postoperative day 1						
Proteinuria	-	-	-	0.609 ± 0.090	0.432–0.786	NS (0.240)
Child-Pugh points	2.438	5	0.786	0.639 ± 0.062	0.479–0.721	NS (0.142)
MELD score	5.947	8	0.653	0.705 ± 0.044	0.620–0.791	**<0.001**
RIFLE	2.684	2	0.261	0.626 ± 0.048	0.531–0.720	**0.007**
SOFA	3.063	8	0.930	0.761 ± 0.043	0.676–0.845	**<0.001**
Proteinuria plus SOFA	7.406	6	0.285	0.828 ± 0.062	0.707–0.949	**<0.001**
Proteinuria plus nonrenal SOFA	11.595	5	0.041	0.823 ± 0.078	0.670–0.977	**0.001**
Postoperative day 7						
Proteinuria	-	-	-	0.757 ± 0.056	0.647–0.866	**<0.001**
Child-Pugh points	6.365	4	0.173	0.750 ± 0.065	0.593–0.847	**0.001**
MELD score	26.161	8	0.001	0.856 ± 0.038	0.782–0.930	**<0.001**
RIFLE	9.602	2	0.008	0.825 ± 0.042	0.742–0.908	**<0.001**
SOFA	6.073	6	0.415	0.899 ± 0.031	0.838–0.961	**<0.001**
Proteinuria plus SOFA	8.625	6	0.196	0.907 ± 0.041	0.825–0.988	**<0.001**
Proteinuria plus nonrenal SOFA	7.856	6	0.249	0.903 ± 0.038	0.828–0.978	**<0.001**
Postoperative day 14						
Proteinuria	-	-	-	0.773 ± 0.067	0.642–0.904	**0.005**
Child-Pugh points	3.469	3	0.325	0.783 ± 0.052	0.682–0.885	**<0.001**
MELD score	134.84	8	<0.001	0.850 ± 0.056	0.740–0.960	**<0.001**
RIFLE	1.658	2	0.436	0.780 ± 0.050	0.681–0.879	**<0.001**
SOFA	24.495	7	0.001	0.892 ± 0.044	0.806–0.978	**<0.001**
Proteinuria plus SOFA	3.987	7	0.781	0.900 ± 0.042	0.819–0.982	**<0.001**
Proteinuria plus nonrenal SOFA	5.009	7	0.659	0.894 ± 0.038	0.819–0.969	**<0.001**

Values in bold are statistically significant (*P*-value < 0.05)

On ICU admission day (before transplantation): The prediction accuracy of the SOFA score was better than those of the Child-Pugh points, MELD score and RIFLE. The proteinuria plus SOFA score has an even better discriminatory power than the SOFA score

On post-transplant day 1, 7, 14: The prediction accuracy of the SOFA and MELD score was better than that of the Child-Pugh points. The proteinuria plus SOFA score has an even better discriminatory power than the SOFA score

Abbreviation: MELD, model for end-stage liver disease; SOFA, sequential organ failure assessment; df, degree of freedom; AUROC, areas under the receiver operating characteristic curve; SE, standard error; CI, confidence intervals; NS, not significant

The proteinuria plus SOFA score [following variables were applied for the calculation: presence of proteinuria (1 point) and SOFA] was defined as the addition of the two variables, with sum ranging from 0 to 25 [4]. The discriminatory ability of this score seemed to be superior to that of other evaluating systems, including proteinuria, CP points, MELD, RIFLE, SOFA, and proteinuria plus nonrenal SOFA scores. AUROC curves were highest for proteinuria plus SOFA on postoperative day 7 to predict 90-day mortality (0.907 ± 0.041). AUROC value for proteinuria plus SOFA determined on postoperative day 1 was significantly higher than that for proteinuria, RIFLE, CP points, and MELD. AUROC for proteinuria plus SOFA determined on postoperative day 7 and 14 were significantly higher than that for proteinuria, RIFLE, and CP points.

### Indices for predicting short-term prognosis

For evaluation and validation of the scoring systems, we compared the sensitivity, specificity, and overall correctness of prediction at cut-off values which could offer the highest Youden index (Table 7). At preoperative, postoperative days 1, 7, and 14, The proteinuria plus SOFA had the best Youden index and overall correctness in predicting 90-day mortality

**Table 7:** Prediction of subsequent 90-day mortality

Predictive factors	Cutoff point	Youden index	Sensitivity (%)	Specificity (%)	Overall correctness (%)
Proteinuria					
On admission	positive	0.16	28	88	58
Postoperative day 1	positive	0.22	64	58	61
Postoperative day 7	positive	0.51	85	66	76
Postoperative day 14	positive	0.55	90	65	77
Child-Pugh points					
On admission	12	0.17	95	22	59
Postoperative day 1	10	0.26	90	37	64
Postoperative day 7	9	0.42	60	82	71
Postoperative day 14	8	0.42	50	92	71
MELD score					
On admission	15	0.19	65	56	61
Postoperative day 1	22	0.27	60	71	66
Postoperative day 7	20	0.58	69	87	78
Postoperative day 14	23	0.62	75	87	81
RIFLE					
On admission	I category	0.04	7	97	52
Postoperative day 1	I category	0.20	57	64	61
Postoperative day 7	I category	0.56	72	82	77
Postoperative day 14	R category	0.60	74	82	78
SOFA					
On admission	5	0.23	52	71	62
Postoperative day 1	11	0.41	77	64	71
Postoperative day 7	7	0.70	83	87	84
Postoperative day 14	7	0.69	85	84	84
Proteinuria plus SOFA					
On admission	5	0.26	57	70	63
Postoperative day 1	12	0.54	79	75	77
Postoperative day 7	8	0.72	85	85	85
Postoperative day 14	8	0.72	89	83	86
Proteinuria plus nonrenal SOFA					
On admission	5	0.26	54	76	63
Postoperative day 1	12	0.54	54	100	77
Postoperative day 7	8	0.68	86	82	84
Postoperative day 14	7	0.67	100	67	84

Optimal cutoff points for predicting 3-month mortality were derived from receiver operator characteristic analysis. On admission (pre-transplant), post-transplant day 1, 7, and 14, the Youden index and overall correctness for predicting 3-month mortality were higher for the proteinuria plus SOFA score than those for the proteinuria, Child-Pugh points, MELD score, RIFLE criteria, SOFA, and proteinuria plus nonrenal SOFA scores

Abbreviation: *MELD* model for end-stage liver disease, *SOFA* sequential organ failure assessment

In this study population, 45 patients had proteinuria while 278 patients had no proteinuria on ICU admission. Patients with proteinuria on admission had higher incidence of AKI (26.8 % vs. 8.8 %, *p* < 0.001), severe infection episodes requiring prolonged courses of antibiotics or inotropic agents (48.8 % vs. 30.7 %, *p* = 0.023), hospital death (31.1 % vs. 10.1 %, *p* < 0.001), and 90-day mortality (37.7 % vs. 10.9 %, *p* < 0.001) than those without proteinuria.

**Fig. 1:**
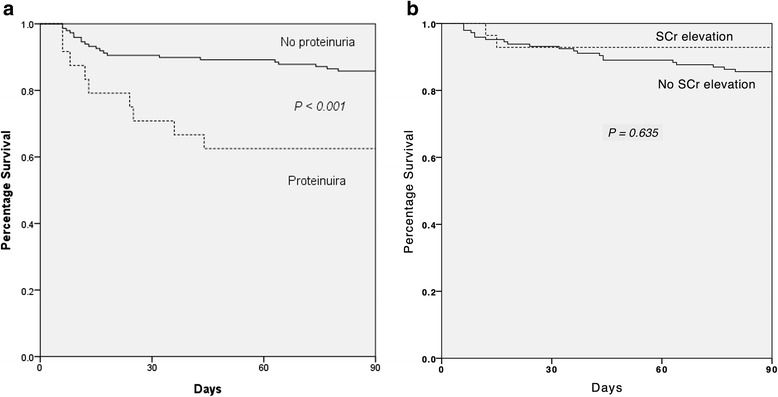
Survival Functions Kaplan-Meier survival analysis in 323 patients according to the data measured before receiving liver transplantation. **a** Cumulative survival rates differed significantly for patients with proteinuria (*n* = 45) and those without proteinuria (*n* = 278) on the first day of ICU admission. **b** Cumulative survival rates did not differ significantly for patients with SCr level elevation (Increase in SCr ≥ 1.5 × baseline) (*n* = 28) and those without SCr elevation (*n* = 295) on the first day of ICU admission. *Abbreviation: SCr, serum creatinine

**Fig. 2:**
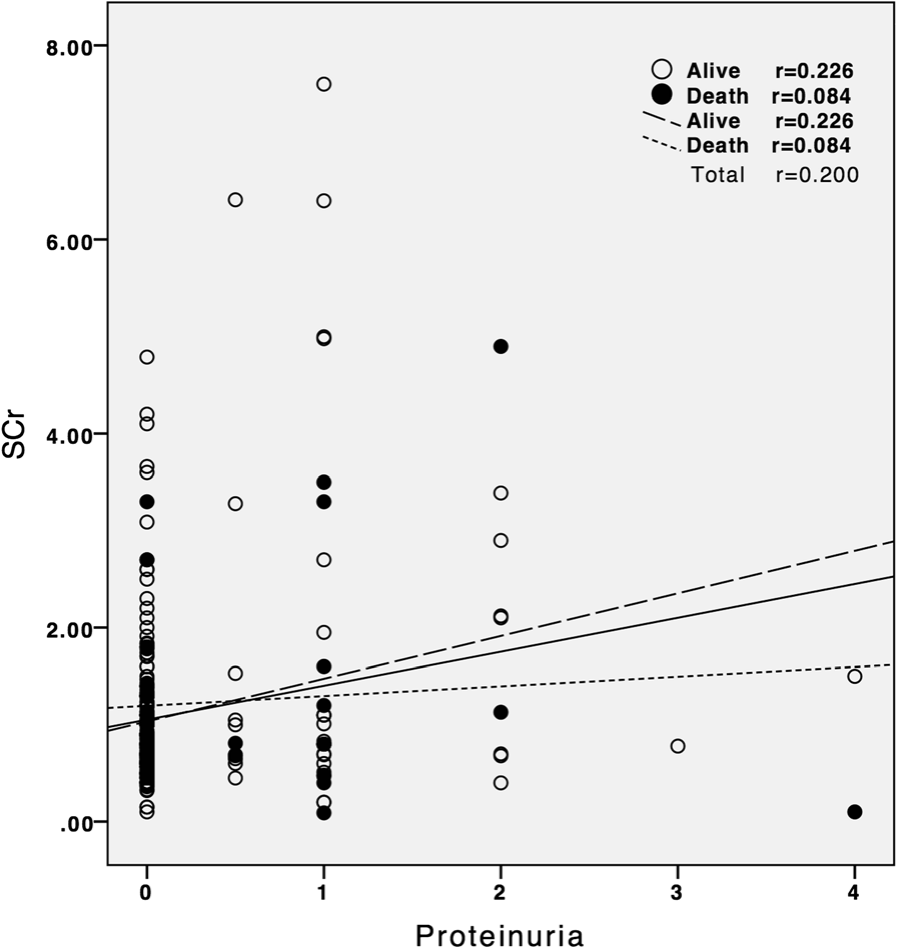
Correlations of proteinuria and SCr measured on the first day of ICU admission for in-hospital mortality. The proteinuria is not correlated significantly (*p* = 0.143) with SCr. *Abbreviation: SCr, serum creatinine

**Fig. 3:**
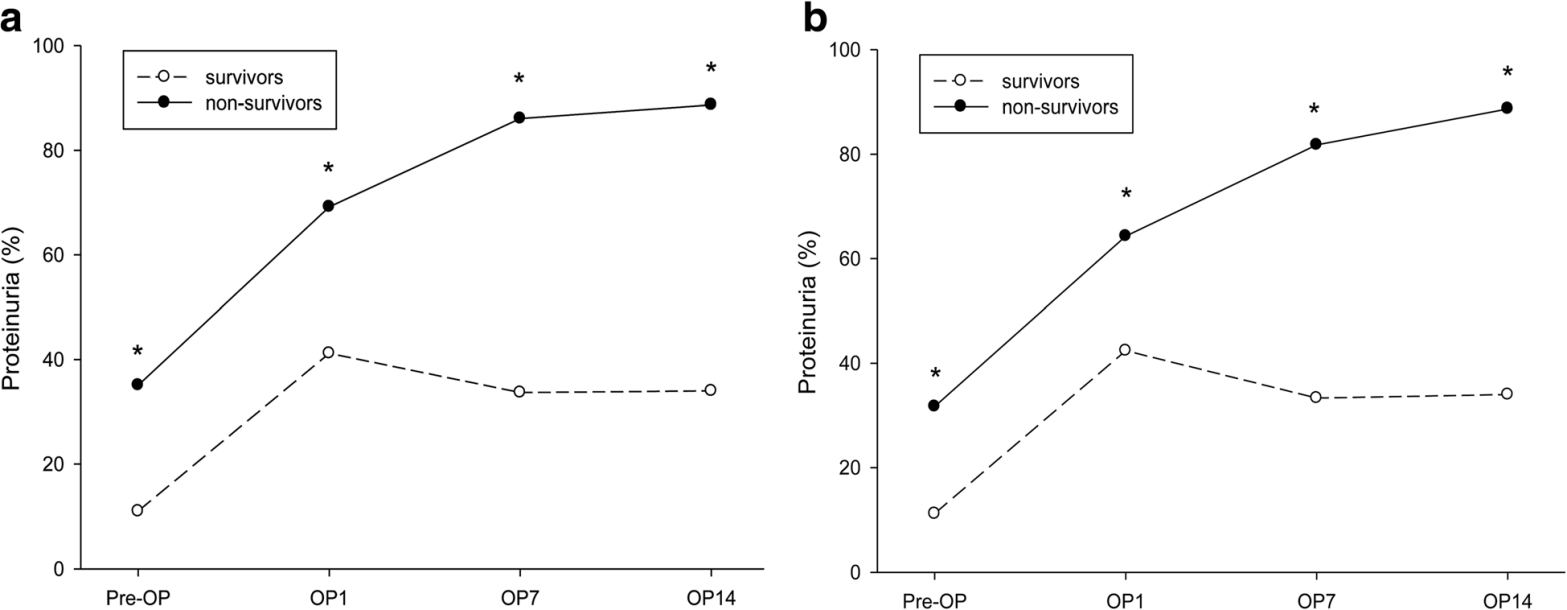
The prevalence of proteinuria for the (**a**) in-hospital survival group (living, *n* = 281) and mortality group (dead, *n* = 42) and (**b**) 90-day survival group (living, *n* = 277) and mortality group (dead, *n* = 46) during the ICU admission day and postoperative days 1, 7, and 14. The prevalence of proteinuria significantly increased during this period among the in-hospital and 90-day mortality groups but not in the survivor groups (* *P* < 0.05)

Figure 1 illuminates the significantly different cumulative survival rates between patients with and without proteinuria as well as the similar cumulative survival rates between patients with and without SCr level elevation (Increase in SCr ≥ 1.5 × baseline) on ICU admission (before transplantation). Fig. 2 shows the weak correlation of proteinuria and SCr measured on ICU admission (*P = 0.143*). Fig. 3 illustrates significant increases in the prevalence of proteinuria during the time (starting before transplantation to 14 days postoperatively) among hospital and 90-day mortality groups but not survival groups.

## Discussion

In the current research, overall hospital survival rate was 87.0 % (281/323), which is in agreement with what had been presented in the literature [8, 18, 19]. Our investigation found that both presence of proteinuria and SOFA score determined on the first day of admission to the ICU were significantly correlated to in-hospital mortality (Tables 2 and 5). Analytical results also proved that discriminatory ability of SOFA was better than those of CP points, RIFLE, and MELD. One notable finding of this study was that presence of proteinuria in combination with SOFA has an even more superior discriminatory power than SOFA alone (Table 6). Moreover, proteinuria plus SOFA also had the highest Youden index and the best overall correctness of prediction (Table 7).

AKI is a common complication in patients with decompensated liver disease, and its occurrence is correlated to poor prognosis [20]. Recently, several promising urinary protein biomarkers have been proved to be remarkably helpful for detecting AKI, such as Calprotectin, neutrophil gelatinase-associated lipocalin (NGAL), cystatin C, interleukin-18 (IL-18), kidney injury molecule-1 (KIM-1), and L-type or liver-type fatty acid-bind protein (LFABP) [2, 21–24]. The presence of protein in urine might reflect structural or functional defects of the glomerular capillary barrier or the reabsorption receptors in the renal proximal tubules. Proteinuria is not only a sensitive indicator but also a risk factor for acute kidney injury [25]. Increasing evidence has indicated that proteinuria itself may activate intrarenal complement cascade, upregulate proximal tubular inflammatory and fibrogenic gene, trigger apoptotic response, and further lead to spreading of renal tubulointerstitial damage and adverse outcomes [26–31]. In the literature, urine albumin to creatinine ratio (UACR) is a precise method for measurement of albuminuria and identification of renal dysfunction [32, 33]. Nevertheless, previous study had documented that preoperative proteinuria could accurately predict the development of AKI in patients undergoing operation, irrespective of it is determined by UACR or urine dipstick analysis [34]. Previous reports also demonstrated that calculating urinary calprotectin/creatinine or NGAL/creatinine ratio does not lead to higher prediction accuracy than using urinary calprotectin or NGAL alone [2, 24]. In this study, proteinuria was detected with the dipstick analysis, the advantages of this examination are inexpensive and easily performed and interpreted. Analyzed data showed presence of proteinuria on ICU admission (before transplantation) was correlated to increased risks of AKI, severe infection episodes, in-hospital mortality, and 90-day mortality (Fig. 1). The occurrence of AKI after transplantation, especially infection related AKI, was correlated to a markedly lower chance of survival (Table 3). Measurement of preoperative proteinuria might be useful to preemptively identify patients who have increased risk of AKI and severe infection episodes. Based on the observed results, it seems that avoiding nephrotoxic agents and choosing therapy carefully are crucial ways of renoprotection for patients present with proteinuria before transplant. More intensive postoperative care and infection prevention strategies, such as more aggressive prophylactic antimicrobial regimens and strict practice for infection prophylaxis, might also help to prevent further adverse outcomes for these patients. Further well-powered research is needed to study this issue.

Among patients undergoing liver transplant, development of renal dysfunction may be attributed to intraoperative caval cross-clamping with vascular outflow obstruction, reperfusion injury, significant infectious event, bleeding, perioperative hypotension with high vasopressor requirement, large-volume transfusions, exposure to nephrotoxic agents or use of calcineurin inhibitors [35–37]. As part of the acute phase response, general vascular permeability increases and the urinary excretion of protein and albumin occur within several hours after operation [38]. In our study, the decreased-donor graft recipients who had prolonged cold ischemia time and the living-donor graft recipients who had longer warm ischemia time, were found to have significantly higher incidences of newly onset proteinuria on the first day after operation. These findings are consistent with previous studies [39–43]. Moreover, the prevalence of proteinuria decreased significantly within 7 days after operation in the in-hospital and 90-day survival groups but not in the mortality groups (Fig. 3). It implies that patients with poor physiologic adaptability are much more intolerable to the transient hemodynamic change of kidney. In addition, the persistence of proteinuria in serial assessment after liver transplantation might represent delayed recovery from the major operation and signify poor short-term prognosis.

Early detection of AKI after liver transplant could potentially retard the progression of renal dysfunction and prevent further adverse outcomes by prompt intervention [36, 44]. Many researches have shown that SOFA can completely present courses of major organ dysfunction and dynamic changes of illness severity after acute insults [45]. We have proposed that SOFA determined on postoperative day 7 has good predictive performance in short-term outcome of patients receiving liver transplant [8]. However, the renal parameter of the SOFA score, SCr concentration, does not seem to elevate until decline of renal function over than 50 %. This means that early detecting AKI by SOFA is hardly possible [36]. Our data revealed that absolute concentration and relative change of SCr levels are not significantly correlated to the presences of proteinuria and patient outcomes (Table 2, Figs. 1, 2), which highlights that SCr concentration is inaccurate for discovering kidney injury among patients with decompensated liver disease [46]. On the contrary, the occurrence of proteinuria might represent subtle renal function changes, and it also signifies generalized inflammatory environment and poor prognosis (correlation between proteinuria and CRP, before operation: *P < 0.001*; postoperative day 1: *P = 0.005)*. In this study, the combination of proteinuria improves the flaws of SCr and the prediction ability of SOFA during perioperative time, particularly on postoperative day 1 (an increase in the AUROC from 0.761 to 0.828) (Tables 6). These exciting findings seem to demonstrate that presence of proteinuria could provide outstanding early prognostic prediction for patients undergoing liver transplantation.

Despite the promising results obtained in this research, there are some possible limitation should be acknowledged. Firstly, this research was performed in just one tertiary medical center, so our results might not be exactly generalized to dissimilar patient population. Secondly, owing to the retrospective nature of our study, not every clinical factor was available. Thirdly, the patient population contained a high proportion of hepatitis B viral infection (34 %) and may present as a special subgroup in the cirrhotic patients. Fourthly, predictive precision of logistic regression models is not perfect and flawless. Finally, the prognostic tools were applied on patients already admitted to the ICU, and were not used as a preadmission screening test, this might skew the analyses.

## Conclusions

To conclude, this study showed that preoperative proteinuria is a significant risk factor of hospital mortality in patients undergoing liver transplantation. For patients exhibiting proteinuria before transplant, we suggest watch out for infection episodes, carefully choose therapy and do prompt intervention to prevent further adverse outcomes. Avoiding prolonged cold or warm ischemia time of transplantation could also reduce organ injury from reperfusion. The presence of proteinuria in serial assessment after liver transplantation has been proven to have early prognostic predictive effect and to assist the SOFA score with better discriminatory power in predicting short-term outcome. For these reasons, we recommend surveying the presence of proteinuria in preoperative and postoperative serial assessment. Proteinuria is supposed to be recognized as an important negative predictor for short-term survival of liver transplant patients.

## Abbreviations

AKI: Acute kidney injury
AUROC: Area under the receiver operating characteristic curve
CP points: Child-pugh points
ICU: Intensive care units
MELD: Model for end-stage liver disease
OP: Operation
RIFLE: The risk of renal failure, injury to the kidney, failure of kidney function, loss of kidney function, and end-stage renal failure
SCr: Serum creatinine
SOFA: Sequential organ failure assessment
TIPS: Transjugular intrahepatic portosystemic shunt
UACR: Urine albumin-creatinine ratio
